# New Options for Subgingival Oral Hygiene with a Flattened Interdental Brush Design – In Vitro Examination and Case Report

**DOI:** 10.3290/j.ohpd.b4043009

**Published:** 2023-04-24

**Authors:** Hans Jörg Staehle, Ti-Sun Kim, Caroline Sekundo

**Affiliations:** a Professor, Clinic for Oral, Dental and Maxillofacial Diseases, University Hospital Heidelberg, Department of Conservative Dentistry, Heidelberg, Germany. Conceptualisation, methodology, investigation, wrote the original draft.; b Professor, Clinic for Oral, Dental and Maxillofacial Diseases, University Hospital Heidelberg, Department of Conservative Dentistry, Heidelberg, Germany. Methodology, edited the manuscript.; c Dentist, Clinic for Oral, Dental and Maxillofacial Diseases, University Hospital Heidelberg, Department of Conservative Dentistry, Heidelberg, Germany. Conceptualisation, data curation, reviewed and edited the manuscript.

**Keywords:** flattened interdental brushes, preventive dentistry, subgingival oral hygiene

## Abstract

**Purpose:**

The subgingival area is only reached to a limited extent during home oral hygiene with the aids available to date. The question was investigated whether a newly developed, flattened cross-sectional design of interdental brushes (IDBs) can extend their subgingival reach.

**Materials and Methods:**

In part I, the passage-hole diameters (PHD) of IDBs of different sizes and side-bristle lengths, with circular and flattened cross-sections, were compared according to the ISO standard 16409/2016. In part II, handling of flattened IDBs was described based on a case report of a patient with generalised stage 4, grade C periodontitis with locally persistent pockets.

**Results:**

Depending on the brush’s size, flattening of IDBs reduced the PHD by 1-18 intervals. IDBs with longer side bristles could thus be inserted into interdental spaces with equal force. This may increase the potential range of IDBs in the vertical dimension. Regular instruction and check-ups are necessary to enable correct handling, as the flattened brushes can only be used in two positions. The observations documented in the case report (duration: 1.5 years) showed that flattened IDBs were associated with reduced signs of inflammation (reduction of pocket depths from 6 to 3 mm, absence of bleeding on probing).

**Conclusion:**

IDBs with a flattened cross-sectional design have not been previously described in the literature. It was shown that flattening of IDBs leads to a size-dependent decrease in PHD. Based on a case report, it was hypothesised that the design change of the IDBs could be clinically relevant in the case of persistent deep pockets in narrow interdental spaces. However, this can only be verified or falsified by clinical studies.

Interdental brushes (IDBs) are important home oral hygiene aids, although by definition they are cosmetic products, not medical devices. They are intended to mechanically affect the ecological niche of the interdental space, which is insufficiently reached by conventional tooth brushing alone.^10,22,23^ In comparison to dental floss and toothpicks, which have traditionally been used for interdental cleaning, IDBs have demonstrated a superior effect on plaque and bleeding scores in a number of studies and systematic reviews.^11,13,15,24-27,30,36,40^ In particular, IDBs have been recommended as a home oral hygiene measure amongst patients with periodontitis.^18^

However, the wide range of products is sometimes confusing, and their handling may be demanding.^33^ IDBs are available in various designs. In longitudinal section, they can be cylindrical, conical, or hourglass-shaped. In cross-section, they are usually circular, but there are also products with triangular incisions.^33,38,39^ Advantages and disadvantages of these different designs have been discussed in a number of studies. Some have found superior performance of hourglass-shaped IDBs,^2,4^ while others have reported equal cleaning effects for cylindrical IDBs.^37^ Likewise, opinions on conical IDBs also differ: While some argue that the latter are easier to handle due to simplified insertion, there are also studies which report less effectivity.^20^ To date, there is no scientific consensus as to which design should be preferred.^6^

Other important characteristics include the length, thickness and density of the radially arranged filaments, which are responsible for the reach, and the internationally agreed upon and standardised passage-hole diameter (PHD) value as a measure of patency at clinically relevant contact pressures during passage.^14^ A PHD range is defined for conical brushes. Mechanical removal of plaque by an IDB can be expected if a certain resistance to passage occurs during its use, i.e. if a brush with an adequate PHD is used.

A review of the currently available product range with particular reference to PHDs was undertaken in 2020 by Sekundo and Staehle.^29^ Tests on the effectiveness of such oral hygiene aids mostly refer to the supragingival areas of the proximal spaces and the adjacent vestibular and oral surfaces.^2-4,16,17,20,25,28,38,39^ There is a dearth of literature on the coverage of subgingival areas, especially in the case of deep pockets.^33^ Satisfactory interdental cleaning is particularly difficult given narrow interdental spaces, especially if combined with persistent deep pockets at the site. It may therefore sometimes be necessary to adapt an IDB’s shape to the individual situation of the patient in order to obtain sufficient contact pressure over the whole area of the interdental space, and at best, to also positively affect the subgingival area. For this reason, we developed a new, flattened cross-sectional design of IDBs, with the intent to improve periodontal parameters in these particular cases.

In part I, the question was addressed as to whether the newly developed, flattened cross-sectional design of IDBs can extend their potential reach into the subgingival space. For this purpose, the in-vitro tests described below were performed. In the second part, the clinical effect of the new design is described on the basis of a case report. This report follows the Case Reporting (CARE) Guideline.^9^

## Part I – In-Vitro Examination

### Materials and Methods

**Fig 1:**
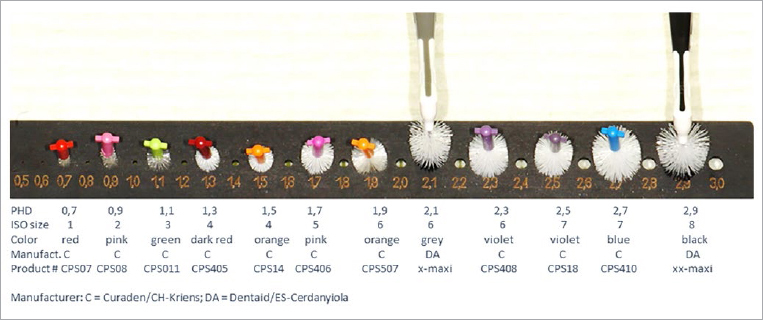
Selection of 12 conventional interdental brushes (circular cross-section) with continuously increasing PHDs, following PHD intervals in steps of two (0.7 mm to 2.9 mm). Values can vary by ~1 to 2 PHD sizes depending on the force applied during passage (especially for larger sizes). For conical brushes, the initial force applied is lower.^33^

**Fig 2:**
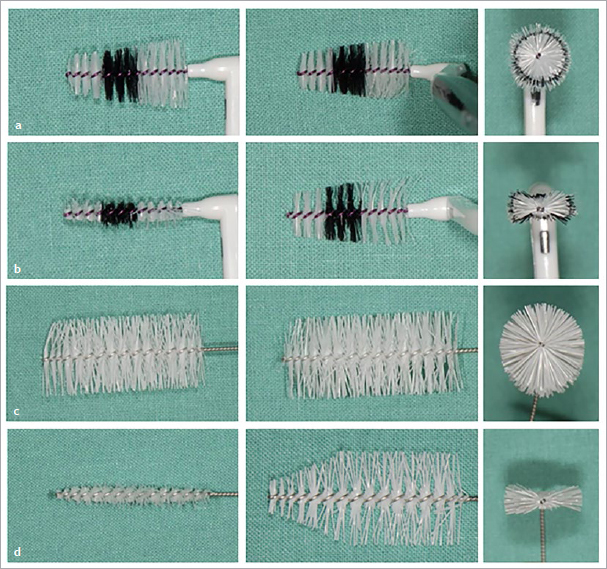
Interdental brushes with circular and flat cross-section. Left: lateral view; center: top view; right: view of each cross-section. (a) Interdental brush with a circular cross-section (mean PHD 2.9 mm) and a maximum side-bristle length of 5.5 mm (xx-maxi, Dentaid). (b) Same interdental brush as (a) only with flattened design resulting in a reduced PHD of eight PHD intervals (mean 2.1 mm). (c) Interdental brush with a circular cross-section (mean PHD 4.2 mm) and a maximum side-bristle length of 7 mm (LS 637, Curaden). (d) Same interdental brush as (c) only with flattened design and angled tip, resulting in a reduced PHD of 18 PHD intervals (mean PHD 2.5 mm).

Fourteen commercially available interdental brushes of different sizes with circular cross-sections (cr) were used to supplement and extend an interdental brush set described in the literature^33^ (Fig 1). The flat versions (f) were produced by two-sided axial cutting and trimming (Fig 2). For two products with cylindrical longitudinal sections (cy), the front surfaces were additionally angled (cn). Flattened brushes were visually checked against a prototype as a standard model in each case. The size of the circular and flat specimens was determined according to ISO standard 16409^14^ using PHDs. This was performed as a size comparison, taking into account the length of the side bristles. Due to the fact that the force that should be applied during PHD measurement (see below) is not specifically stated in the ISO standard, but merely referred to as “clinically relevant”,^14^ the examinations were performed by 10 examiners in randomised order, and intra- and interexaminer reliability were calculated. Ten dentists were calibrated in individual sessions, during which the content of the ISO standard was explained and the procedure it specifies was demonstrated.

The 2.0 ± 0.1-mm-thick measurement plate required for PHD determination had holes with diameters continuously increasing in 0.1 mm increments. Eight specimens were inserted in each case in descending order. The test was terminated as soon as the smallest hole was reached, at the passage of which none of the specimens showed any deformation at “clinically relevant” force (according to the ISO specification).^14^ Four weeks after initial calibration, the examiners were asked to repeat the examination. Intra- and interexaminer reliability was tested by the intraclass correlation coefficient (ICC) for absolute agreement (unadjusted model). Values above 0.9 were considered to represent very good clinical reliability.^19^ The statistical significance level was set at p ≤ 0.05.

### Results

The average measured PHD sizes of the original brushes and the flat modification are shown in Table 1. The new design reduced the PHD in all cases. The reliability analyses revealed high intra- and interexaminer reliability (Table 2). The intraexaminer reliability was 0.999 (ICC) for both forms of IDBs.

**Table 1:** PHD measurements of the original brushes and the flat modifications (mean values from both times of measurement)

Product	PHD, original product (cr)		PHD, flat modification (f)		Size difference		Max. side-bristle length in mm	Longitudinal profile
	Mean	SD	Mean	SD	Mean	SD		
CPS06, C	0.64	0.05	0.53	0.05	0.11	0.08	1.1	Cy
CPS06, C	0.64	0.05	0.50	0	0.14	0.05	1.1	cy/cn
CPS07, C	0.70	0	0.6	0.02	0.09	0.05	1.25	cy
CPS08, C	0.89	0.04	0.7	0.00	0.19	0.02	1.6	cy
CPS011, C	1.06	0.05	0.81	0.02	0.25	0.00	2.5	cy
CPS405, C	1.40	0.10	1.03	0.06	0.37	0.02	2.5	cy
CPS14, C*	1.44	0.09	1.07	0.05	0.37	0.06	2.5	co
CPS406, C	1.76	0.08	1.30	0.05	0.46	0.05	3.25	cy
CPS507, C	1.85	0.06	1.38	0.06	0.47	0.05	3.75	cy
x-maxi soft, DA*	2.04	0.07	1.53	0.07	0.52	0.06	4.5	co
CPS408, C	2.34	0.08	1.70	0.08	0.65	0.07	4.0	cy
CPS18, C	2.50	0.09	1.81	0.08	0.69	0.08	4.0	cy
CPS410, C	2.80	0.17	2.01	0.10	0.78	0.08	5.0	cy
xx-maxi, DA*	2.88	0.09	2.11	0.13	0.77	0.12	5.5	co
LS 637, C	4.24	0.23	3.03	0.13	1.22	0.19	7.0	cy
LS 637, C	4.24	0.23	2.47	0.17	1.77	0.28	7.0	cy/cn

Manufacturer: C = Curaden; Kriens, Switzerland. DA = Dentaid; Cerdanyiola del Vallès, Spain. Cross-sectional profile: cr = circular; f = flat. Longitudinal profile: cy = cylindrical; co = conical; cn = cylindrical and narrow tip. *Since conical interdental brushes may have a size spectrum, the PHD measurements refer to the size at which the conical brush passes completely, according to ISO classification.

**Table 2:** Intra- and interexaminer reliability (n = 10)

	ICC	95% confidence interval		F-test with true value 0			
		lower limit	upper limit	Value	df1	df2	p-value*
**Interexaminer reliability measurement of original interdental brushes**							
Observation time (0 M)	0.999	0.998	1.0	1091.802	13	117	<0.001
Observation time (1 M)	0.999	0.998	1.0	1437.560	13	117	<0.001
1st + 2nd observation time	0.999	0.999	1.0	2362.745	13	247	<0.001
**Interexaminer reliability measurement of modified interdental brushes**							
Observation time (0 months)	0.999	0.998	1.0	1074.999	13	117	<0.001
Observation time (1 month)	0.999	0.998	1.0	1230.293	13	117	<0.001
1st + 2nd observation time	0.999	0.999	1.0	2594.887	13	247	<0.001
Intraexaminer reliability (1st + 2nd observation time): mean ± SD	0.998 ± 0.001	0.994 ± 0.005	0.999 ± 0.001	1127.777 ± 1347.338	13	0	<0.001

*Statistical significance set at p≤0.05.

## Part II – Case Report

The case presented here reports on a 49-year-old male patient. He was given detailed verbal and written information as well as access to records and images, and provided written informed consent.

The patient is currently largely symptom-free. He presents at periodontal follow-ups in the course of his supportive periodontal therapy. The initial diagnosis was generalised periodontitis stage 4, grade C, modified by diabetes mellitus and smoking. During the course of supportive periodontal therapy, the patient was mostly stable but showed localised infections at some sites. He has had insulin-dependent diabetes (type 1; current HBA1c: 8.0%) for more than 20 years. The patient continuously monitors his blood glucose levels with a sensor and adjusts the self-injected insulin doses to his dietary habits. He has been a smoker for about 20 years (8.5 pack-years).

### Dental History

**Fig 3:**
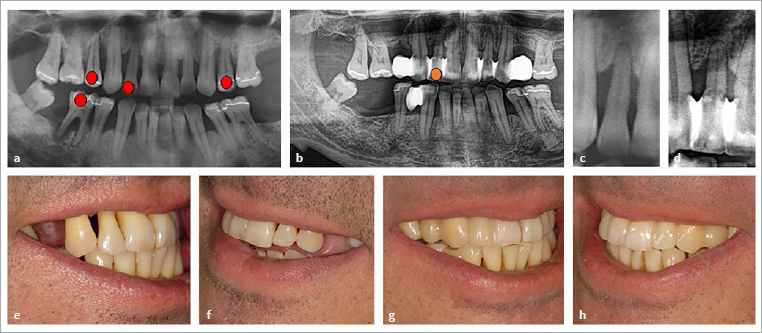
(a) Panoramic radiograph of the presented patient with generalised periodontitis, stage 4, grade C (rapidly progressing) at the age of 42 years. Unfavourable prognosis of the teeth marked in red. (b) Situation after 6 years (patient age: 48 years). After systematic periodontal therapy including surgical therapy, some teeth with questionable prognosis could be preserved. However, three teeth were extracted. In some areas, non-prep bridges were inserted. The mobile lateral incisors were splinted with composite. The prognosis of the maxillary right lateral incisor (orange dot) remains doubtful due to advanced bone loss. (c) and (d) An enlarged view of the maxillary right lateral incisor shown in (a) and (b) shows no significant changes in bone loss between baseline (c) and the situation after six years (d). (e) and (f) Clinical situation after extraction of the maxillary second premolars. (g) and (h) Clinical situation after insertion of non-prep bridges and splinting with simultaneous shape correction of the lateral incisors.

After the initial diagnosis seven years ago, the patient received active periodontal treatment according the guidelines at the time of treatment. Afterwards, the patient received supportive periodontal treatment. Supportive periodontal therapy with professional mechanical plaque removal was performed on all teeth a total of 21 times after the initial anti-infective therapy. Scaling and root planing was performed five times on various teeth, including twice on the maxillary right lateral incisor (most recently 5.5 years ago). Flap surgery was performed twice in the posterior region due to persisting deep pockets in anatomically challenging situations, but not on the maxillary right lateral incisor, in order to avoid unfavourable aesthetic outcomes. Both maxillary second premolars with endodontic-periodontal lesions had to be extracted during the course of therapy. The gaps were closed with non-invasive methods (non-prep bridges), in keeping with the concept of “frugal” dental care.^31,32,34^ In addition, non-invasive splinting and reshaping of the maxillary anterior dentition was carried out with directly applied composite. This not only stabilised and improved the aesthetic situation, but also created better counter-bearing for the use of IDBs (Fig 3). It is very important to take particular care when performing this type of splinting (i.e. leaving enough space for interdental cleaning aids and ensuring smooth margins), and the patient must be instructed in the use of interdental brushes; otherwise, this measure can have a detrimental effect on home oral hygiene.

After performing systematic periodontal treatment, there has been a decrease in periodontal probing depths. In general, probing depths are ≤ 3 mm without bleeding on probing. However, despite the above-mentioned measures, probing depths of up to 5-6 mm and bleeding on probing remained on a few teeth, such as the maxillary right lateral incisor.

Since periodontal nomenclatures, classifications, and treatment regimens have varied over the years, the information in this paper focuses on commonly accepted risk factors, diagnoses according to the current nomenclature^35^ and summary listings of nonsurgical and surgical periodontal therapy at various stages (from initial interventions to supportive periodontal therapy). The patient has been regularly provided with detailed information, instruction and motivation for home plaque control measures during the different phases of therapy.

### Clinical Findings

At the first appointment, the patient was 42 years old. Extra-orally, no pathological abnormalities were observed. The intraoral findings are given below.

Twelve teeth were missing, the mandibular right first molar had just been extracted. On the remaining 22 teeth, the dental hard tissues were caries-free. Moderate non-caries related changes such as abrasions, attritions and erosions were evident. The patient had 9 intact restorations. There was no evidence of endodontic disease. Function and appearance were also not seriously affected. With only a few exceptions, the periodontal examination at the time revealed generalised increased probing depths of up to 10 mm and attachment loss of up to 11 mm (based on 6 measurements per tooth in each case). The diagnosis was generalised periodontitis stage 4, grade C, modified by diabetes mellitus and smoking. There was no alternative differential diagnosis.

The gingival bleeding index (GBI according to Ainamo and Bay) was 0%, and the plaque control record (PCR according to O’Leary) was 52%. Generalised bleeding occurred after probing. The situation on the maxillary right lateral incisor, focused on in this case report, is listed in Table 3. After seven years, the now 49-year-old patient showed generalised probing depths of 2-4 mm and absence of bleeding on probing (see above), with a few localised exceptions. The GBI has fluctuated between 0-3% and the PCR between 15%-47% during the years of supportive periodontal therapy. Although the direct splinting and reshaping measures improved the use of IDBs, increased probing depths up to 6 mm and bleeding on probing remained on the maxillary right lateral incisor. Details are listed in Table 3.

**Table 3:** Probing depth and attachment level on the maxillary right lateral incisor over time

Vestibular attachment level: Initial findings 7 years later 8.5 years later (1.5 years after use of flat brushes)	Disto-vestibular 9 mm Disto-vestibular 9 mm Disto-vestibular 8-9 mm	Vestibular 9 mm Vestibular 9 mm Vestibular 8 mm	Mesio-vestibular 7 mm Mesio-vestibular 8-9 mm Mesio-vestibular 8-9 mm
Oral attachment level: Initial findings 7 years later 8.5 years later (1.5 years after use of flat brushes)	Disto-oral 9 mm Disto-oral 9 mm Disto-oral 9 mm	Oral 9 mm Oral 9 mm Oral 9 mm	Mesio-oral 9 mm Mesio-oral 9 mm Mesio-oral 9 mm
Vestibular probing depth: Initial findings 7 years later 8.5 years later (1.5 years after use of flat brushes)	Disto-vestibular 6 mm Disto-vestibular 6 mm Disto-vestibular 3 mm	Vestibular 3 mm Vestibular 2 mm Vestibular 1-2 mm	Mesio-vestibular 3 mm Mesio-vestibular 5-6 mm Mesio-vestibular 3 mm
Oral probing depth: Initial findings 7 years later 8.5 years later (1.5 years after use of flat brushes)	Disto-oral 7 mm Disto-oral 5 mm Disto-oral 4 mm	Oral 6 mm Oral 4 mm Oral 4 mm	Mesio-oral 6 mm Mesio-oral 5 mm Mesio-oral 3 mm

HBA1c levels varied over the years, depending on lifestyle habits. At the beginning, they were over 10%. In the following years, they fluctuated between 7.1% and 9.3%. Smoking habits did not decrease; rather, they increased especially during stress. Cessation efforts were unsuccessful.

In the course of supportive periodontal therapy, variable clinical findings occurred on the maxillary right lateral incisor, but overall, there was little change. This corresponds with the radiograph taken six years after baseline (Fig 3). At the mesio-vestibular surface, probing depths actually increased to 6 mm compared to baseline findings of 3 mm, and attachment levels reached 8-9 mm compared to the baseline findings of 7 mm.

As far as the measurements of the attachment levels are concerned, however, it must be taken into consideration that a comparison with the initial situation was difficult due to the composite splinting and reshaping that took place later, the boundaries of which could hardly be determined exactly in the gingival region. The size of the interdental spaces (determined on the basis of PHD) was approximately 2.9 mm between the maxillary right lateral incisor and adjacent teeth.

The patient had shown a high degree of therapy adherence in the past. He had previously used individually selected IDBs with a circular cross-section and was very familiar with the use of these oral hygiene aids. The correct sizes and use were re-evaluated regularly during supportive periodontal therapy. Based on the findings on the maxillary right lateral incisor, intervention was deemed necessary. There was no clear answer to the question of why the tooth had persistently increased probing depths. It was assumed that adequate oral hygiene was limited there due to special anatomical conditions. Overall, an interaction of risk factors (diabetes mellitus and smoking) on the one hand and localised limited plaque control on the other hand may have played a role. In view of the previous generally successful periodontal therapy, the prognosis for the maxillary right lateral incisor was classified as moderate to unfavourable, but not as hopeless.

### Therapeutic Interventions

The aim of the treatment was to reduce probing depths and bleeding on the maxillary right lateral incisor. Overall, no changes were made to the patient’s previously established follow-up procedures, nor were any additional periodontal interventions performed, as these treatment modalities were considered exhausted. The only change involved a minor modification of home oral hygiene by customiing the shape of IDB to be used there (trimming to a flat design). The aim was to enable the patient to better reach the subgingival area, which is evidently difficult to access. In order to find suitable sizes, product tests were carried out (see Part I).

As part of the switch from circular to flat IDBs on the maxillary right lateral incisor, flattened products with a PHD of approximately 2.1 mm were initially used (xx-maxi, Dentaid; Cerdanyiola del Vallès, Spain) so that the patient could easily practice handling them with low passage resistance. The patient was asked to report immediately in case of gingival irritation and/or pain. The patient, who had been instructed in detail in the use of the flattened brushes, was recalled after a few weeks for follow-up examinations in order to check for trauma. Once correct handling was ensured, the patient was switched to flattened IDBs with a PHD of approximately 2.5 mm (LS 637, Curaden; Kriens, Switzerland) on the maxillary right lateral incisor. After up to 17 months, control examinations were performed on the tooth area in focus with regard to the condition of the gingiva, the level of probing depths and bleeding on probing.

**Fig 4:**
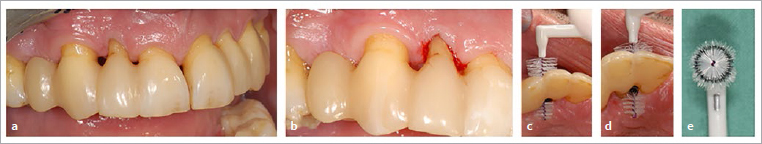
(a) and (b) Situation of the maxillary right lateral incisor after 7 years (patient age now 49 years). Clinical inspection revealed papillary compression, which was more pronounced distally than mesially. Distal and mesial surfaces showed probing depths of up to 6 mm with bleeding on probing. (c) to (e) The maximum interdental size (measured by means of PHD) was approximately 2.9 mm. Use of an interdental brush with a PHD of approximately 2.9 mm and a side-bristle length of maximum 5.5 mm (xx-maxi Dentaid).

**Fig 5:**
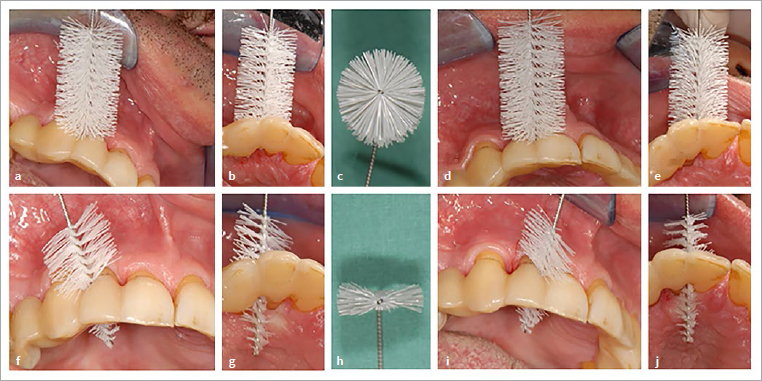
Trial fitting of IDBs with longer side bristles. (a, d, f, i) buccal view; (b, e, g, j) incisal view; (c, h) view of the cross-section of the IDB used. (a–c) Distal interdental space: IDB (with round cross-section, PHD of ~4.2 mm, side-bristle length of 7 mm) cannot be inserted. (f–h) Same IDB as in (a–c), but flattened, PHD now ~2.5 mm, passes with moderate force. (d, e, i, j) Analogous situation mesially.

**Fig 6:**
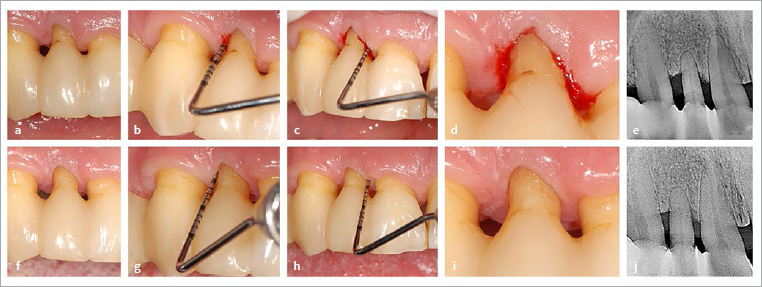
(a) to (e) Depiction of details of the maxillary right lateral incisor, now 7 years after initial presentation (patient age now 49 years) showing mesio- and distovestibular probing depths of 6 mm with bleeding on probing. The patient previously used IDBs of different sizes with circular cross-section. (f) to (j): After switching to a flattened IDB with a potential initial reach a maximum of 5.5 mm and later a maximum of 7.0 mm, the following occurred within a period of approximately 1.5 years (17 months): reduction in mesio- and disto-vestibular probing depths to 3 mm, as well as disappearance of bleeding on probing (note: radiographic control (j) occurred after only 8 months). Possibly, the flattened IDB with the long side bristles was able to reach further into the periodontal pocket than the circular brush. The reduction in probing depths in the now 50.5-year-old patient seems to reflect further gingival recession.

The patient attended his appointments regularly and reliably. The flattened interdental brushes were changed approximately every four weeks. After 6 months, significant improvements were visible, which had further stabilised by the follow-up after about 1.5 years (17 months). Figures 4 and 5 show the periodontally damaged maxillary right lateral incisor of the 49-year-old patient. Prior to the use of flat IDBs, this tooth had exhibited approximal probing depths of up to 6 mm and bleeding on probing despite all treatment measures, supportive periodontal therapy with short recall intervals and thorough oral hygiene, including the use of circular IDBs selected by dental personnel (see above). Seventeen months after the use of flat IDBs, a decrease in probing depths of 3 mm (maximum probing depths now 3 mm), and bleeding on probing had disappeared in the now 50-year-old patient (Fig 6, Table 3). It must be noted that the patient was informed about the purpose of the new oral hygiene aid; thus, a certain risk of bias due to behavioural changes (i.e. more regular use of brushes than before) cannot be ruled out.

PHD size measurements on the patient’s interdental spaces were performed in such a way that successively larger IDBs were introduced until they just allowed passage with moderate resistance. The flattened IDBs were slightly easier to pass after 17 months of use, but the PHDs had not changed. The gingival surface appeared smooth under magnification in the adjacent interdental spaces before the change to flat IDBs. After 1.5 years, it appeared slightly stippled, indicating increased keratinisation. The distance between the gingival margin and the cementoenamel junction was approximately 2-3 mm higher than before. This indicates that the reduction in probing depths was predominantly due to gingival recession rather than attachment gain. After the use of flattened IDBs, the patient presented with discrete gingival irritations (redness) at his first check-up. The instructions for proper use were repeated. Apart from the initial minor irritation of the interdental areas, no further pathological signs were detected. The stippling largely remained unchanged. The patient was asked to report any adverse reactions immediately. No further irritations were detected during further controls.

## Discussion

Two parameters are of particular importance for subgingival cleaning using IDBs: potentially high reach, if possible also vertically (realisable by long side bristles); sufficiently high contact pressure (achievable through adequate PHDs) with comfortable insertion.

According to a study by Sekundo and Staehle, IDBs with circular cross-sections are commercially available within a PHD spectrum of approximately 0.6 mm to 5.2 mm.^29^ An assortment presented in the literature for routine dental practice, which follows PHD intervals in steps of two, comprises 12 IDBs with a spectrum of approximately 0.7 mm to 2.9 mm^33^ (Fig 1). The differentiation criterion here is not primarily the clinical indication, but the size system. Since no exact insertion force is specified in the corresponding ISO standard when determining PHDs, measured values can vary, depending on the force applied. This can be observed especially for products with larger PHDs (see standard deviations in Table 1).

In the present study, a PHD range of 0.64 mm to 4.24 mm was recorded in circular cross-section. The length of the side bristles varied from 1.1 mm to 7.0 mm. Since the wire core of an IDB is positioned approximately equigingivally, the largest IDBs currently available have a potential reach of a maximum of 7 mm into a periodontal pocket, depending on the nature of the interdental soft tissue, the probing depth as well as other factors, such as the surface friction of the devices. However, we could not assess how far the bristles extend below the gingival margin in reality, and could only observe the clinical outcomes.

Regarding the dimensions of the flattened prototypes, it must be noted that slight alterations may have occurred due to manual trimming. These are mirrored in the standard deviations obtained from the subsequent PHD measurements (Table 1). As these remained low, however, this seems to be negligible as a source of error. The smallest circular IDB with a PHD of 0.64 mm achieved a PHD of 0.50 mm by flattening and reducing the tip. However, it seems questionable whether this is clinically relevant at such dimensions. The situation is different for the large PHD. The largest circular IDB with a PHD of 4.24 mm was assigned a PHD of 2.47 mm (difference = 18 sizes) after flattening. If a reach of 7 mm was desired in the case of a deep pocket but the PHD was less than 4.24 mm due to the given anatomical conditions, the available IDB with a side-bristle length of 7 mm could not be inserted. Only by flattening would it be possible to pass through narrower interdental spaces, namely up to a PHD of 2.47 mm. If the PHD is less than 2.47 mm, other commercially available IDBs could be used, but so far, only products with a circular cross-section and a side-bristle length of up to 5.5 mm are available for these areas. Flattening would allow the passage of even narrower interdental spaces down to PHD of 2.11 mm without restricting the side-bristle length. This means that flattening provides a brush design that has the potential to reach narrow interdental spaces with deeper pockets. However, the conversion from circular to flat also changes the handling. While a single position per interdental space is often sufficient with the circular design, two positions per interdental space are available with the flat design. The use of flattened interdental brushes should thus be restricted to patients who are familiar with interdental brushes, having received in-depth instructions, and supervised by dental personnel by at regular check-ups.

No literature is available on the clinical use of flattened IDBs. The following aspects should be considered with regard to the anatomical structures involved: If the patient has no increased periodontal pockets, IDBs are likely to move between root surfaces and coronally located gingival tissue. Areas extending further subgingivally are more difficult to assess. The type of tissue with which the flexible side bristles of an interdental brush come into contact is not precisely known. The wire core may also come into lateral contact with the root surface; however, there is no evidence on this subject to date. Again, we thus recommend that these brushes be used by properly instructed patients only.

Regarding the vertical passage of rigid periodontal probes, it is assumed that under healthy periodontal conditions, the probe tip stops approximately 0.4 mm coronal to the apical end of the marginal epithelium. However, in the case of periodontal inflammation with pathologically altered pocket epithelium, the probe tip penetrates 0.3-0.5 mm apically of the coronally located epithelial attachment at a probing depth of up to 5.5 mm, for example (CPI grade 3).^7,21^ It is questionable whether, at a vertical probing depth of 7 mm, an IDB with a side-bristle length of 7 mm will reach the same anatomical structures as the probe. Even with sufficient contact pressure, there is likely to be a residual area that is not fully accessed due to “evasive movements”of the flexible bristles. What effect the side bristles have subgingivally is also not exactly known. Possible effects could include the disruption of the microflora, changes to the biofilm, loosening of plaque or mechanical irritation. It can only be assumed that by flattening the cross-sectional design, the subgingival reach can be increased with the same passage resistance compared to round IDBs, if products with long side bristles are selected while ensuring sufficiently high PHDs during passage.

The first clinical observations suggest that the use of brushes with the longest possible individual reach (maximum available side-bristle length) with sufficient contact pressure (use of the highest determined PHD) could favour a reduction of inflammatory signs (reduced probing depths, decrease in probing bleeding). This leads to the hypothesis that in the case of persistent deep pockets in narrow interdental spaces, regular home use of flat IDBs may lead to positive effects. This new type of interdental brush may also be able to act as a “vehicle” to transport drugs or antimicrobial agents closer to the site of inflammation.

Regarding clinical relevance, the following should be noted: Despite correct interventions and good patient adherence, localised persistence or recurrence of pathological deep pockets with bleeding on probing may occur after anti-infective therapy. To counteract this, among other things the literature recommends re-instrumenting the affected areas subgingivally, applying topical medications and/or performing surgical measures, for example, open curettage.^8,18^ More invasive surgical interventions (such as gingivectomies) may, however, be associated with hypersensitivity and aesthetic disadvantages. A shortening of recall intervals is also recommended.^5^ These treatment options also do not always lead to success. This type of patient case is illustrated by the presented casuistic: conservative treatment and retreatment options had been exhausted, more extensive surgical interventions could have been envisaged and were performed on other teeth, but were avoided on the lateral incisor due to an unfavourable risk-benefit analysis of resulting aesthetics. Moreover, stabilisation of the patient’s periodontitis was probably negatively influenced by systemic factors (smoking history and type I diabetes mellitus), as well as by the unfavourable anatomical characteristics of the narrow interdental space. In such situations, additional products, such as the flattened IDBs presented here, could be helpful as a supplement to the intervention spectrum, in order to maintain the function of the affected teeth for as long as possible.

Despite the frequently expressed concern that interdental brushes can lead to traumatic damage, there is little scientific evidence on the subject to date. However, as it has been reported that traumatic toothbrushing can cause soft tissue damage,^1,12^ and the same potential is probably present for interdental brushing. To the best of our knowledge, there are no studies on hard tissue lesions caused by interdental brushes, and as the latter are used without toothpaste and thus without abrasives, the risk can probably be considered low. Initial clinical experience shows that a patient must be well instructed and trained in the use of flat IDBs in order to allow optimal placement of the product (with two positions per interdental space) and avoid injury. The aim is to keep probing depths stable despite unfavourable anatomical conditions and to avoid bleeding on probing in the long term. The patient was able to cope with the IDBs and was satisfied that the probing depths could be reduced and that there was no bleeding on probing. The reported increased keratinisation of the gingiva and gingival recession can be accepted in exchange for the reduction of periodontal inflammation. Systematic studies are needed before wider use is recommended, on the basis of which an improved risk-benefit assessment can be made.

## Conclusion

On the basis of this case report, it is hypothesised that design changes of IDBs (here: flattening in cross-section) may be clinically relevant in certain cases of therapy-resistant periodontal pockets. Clinical studies should be undertaken to test this hypothesis further.
